# Identification of m6A-Related lncRNA to Predict the Prognosis of Patients with Hepatocellular Carcinoma

**DOI:** 10.1155/2022/4169150

**Published:** 2022-05-09

**Authors:** Hao Yang, Hong Yang, Wei Zhang, Juping Wang, Liping Sun, Juan Gao, Haiping Zhao, Zhenfei Wang

**Affiliations:** ^1^Department of Radiation Oncology, Inner Mongolia Cancer Hospital & Affiliated People's Hospital of Inner Mongolia Medical University, Huhhot 010020, China; ^2^The Laboratory of Radiation Physics and Biology, Inner Mongolia Cancer Hospital & Affiliated People's Hospital of Inner Mongolia Medical University, Huhhot 010020, China; ^3^Department of Oncology, Inner Mongolia People's Hospital, Huhhot, Inner Mongolia, 010017, China; ^4^Department of Abdominal Tumor Surgery, Affiliated Hospital of Inner Mongolia Medical University, Huhhot 010020, China; ^5^The Laboratory for Tumor Molecular Diagnosis, Inner Mongolia Medical University, Huhhot 010020, China

## Abstract

Hepatocellular carcinoma (HCC) is the third leading cause of cancer-related death worldwide. In the past decades, HCC treatment has achieved great progress; however, the overall prognosis remains poor. Therefore, it is the need of the hour to identify new prognostic biomarkers which can advance our understanding related to the underlying molecular mechanism of adverse prognosis and apply them to clinical work in prognosis prediction. In the present study, data of 576 HCC patients and 292 normal control cases from TCGA and ICGC databases were enrolled to our bioinformatic analysis. SNHG1 and SNHG3 were identified as overlapping genes in TCGA and ICGC databases using Pearson correlation analysis and univariate Cox regression analysis. Further, we used the median of the SNHG1 and SNHG3 expression values as the cutoff values to define the HCC patient groups with high or low expression level. The subsequent analysis revealed that abnormal high expression of SNHG1 or SNHG3 affected the immune infiltration patterns and the crosstalk among immune cells. Moreover, high expression of SNHG1 or SNHG3 resulted in drug resistant to AKT inhibitor VII, bexarotene, bicalutamide, dasatinib, erlotinib, and gefitinib. In addition, lower tumor neoantigen burden was observed in high SNHG1 or SNHG3 group. Further, we found significant relation between the aberrant upregulation of SNHG1 and SNHG3 in tumor grade and stage. We established a nomogram to systematically predict the 5- and 8-year overall survival of liver cancer patients with good accuracy. Finally, the in vitro assays suggest that SNHG1 and SNHG3 promote the proliferative, migratory, and invasive abilities of HCC cells.

## 1. Introduction

Hepatocellular carcinoma (HCC) is the sixth most common tumor and the fourth leading cause of cancer death throughout the world [1]. HCC accounts for >80% of primary liver cancer with rapidly increasing incidence [2]. The tumorigenesis of HCC is complicated, and the major risk factor is related to chronic hepatitis viral infections such as hepatitis B or hepatitis virus [3, 4]. Moreover, additional cases are observed from alcohol abuse, afatoxin B1 intoxication, and genetic diseases. With the recognition of the severity and high mortality, great progress has been achieved in the HCC treatment in recent years. The survival rate for HCC has been improved. However, the overall prognosis remains far from satisfactory. The prognosis for HCC is poor due to the high recurrence, distant metastasis, and limited treatment options. This makes it imperative to advance our understanding related to underlying molecular mechanism of tumorigenesis and progression of HCC, which can enhance the overall prognosis of HCC patients.

N6-methyladenosine (m6A) is the methylation at the nitrogen-6 position of the adenosine base. It is the most abundant internal modification of mRNA and long noncoding RNA (lncRNA) in eukaryotes with the feature of dynamic and reversibility [5–7]. The m6A modification of RNA is highly conserved and prevalent in eukaryotes [7, 8]. Accumulating evidences have revealed that m6A methylation could regulate RNA stability, splicing, export, and translation [9]. Moreover, m6A methylation participates in diverse biological processes and also has been observed to regulate gene expression.

m6A modification is regulated by the dynamic interaction of methyltransferases (also known as “writers”), binding proteins (also known as “readers”), and demethylases (also known as “erasers”). Recent studies have shown that m6A modification and m6A regulators played critical role in tumor oncogenesis and progression in multiple cancers including HCC [10–12]. m6A modification has been observed to decrease in HCC tissues compared to the adjacent normal tissues and is involved in HCC metastasis [10]. METTL3 is the core member of classical complex of “writers,” promotes HCC progression in m6A dependent manner [13], and acts as potential prognostic biomarkers in HCC [13]. WTAP is another member of the classical complexes of “writers,” guides m6A modification, and facilitates the progression of HCC HuR-ETS1-p21/p27 axis [14]. Whereas YTHDF2 is the first identified m6A “reader” involved in suppressing HCC cell proliferation and tumor growth and metastasis [15, 16]. FTO, which is the earliest discovered m6A demethylase, plays oncogenic role in HCC via demethylation of PKM2 [17]. Moreover, emerging evidences indicated that m6A modification was associated with the therapeutic resistance of tumors. For instance, m6A modification is related to antichemotherapy and radiotherapy sensitivity of cancer cells [18, 19]. HNF3*γ* reduction mediated by m6A methylation renders HCC resistant to sorafenib therapy [20]. Similarly, METTL3-mediated m6A modification of FOXO3 was reported to regulate sorafenib resistance.

According to previous studies, lncRNAs are aberrantly expressed in multiple types of cancers including HCC [21–25]. Further, numerous evidences have shown involvement of lncRNAs in oncogenesis and progression of HCC. Additionally, lncRNAs are closely linked to prognosis. lncRNA MCM3AP-AS1 acts as an oncogenic lncRNA and is positively associated with advanced tumor stage, high tumor grade, and poor prognosis of HCC patients [13]. lncRNA-PDPK2P plays a key role in HCC development and progression. Moreover, it can serve as a molecular target to predict the prognosis of HCC patients [26]. Further, previous studies reported a decreased level of lncRNA-D16366 in tissues and serum of HCC patients and can be an independent diagnostic and potential prognostic indicator of HCC [27]. Whereas lncRNA ROR1-AS1 was observed with significant increment in HCC patients and may act as a biomarker for the prognosis [28].

In the present study, data from a large cohort of HCC patients and the normal control cases from TCGA and ICGC databases were enrolled to screen m6A-related prognostic lncRNA. In the present study, we identified 2 m6A-related prognostic lncRNAs, small nucleolar RNA host gene 1 (SNHG1), and small nucleolar RNA host gene 3 (SNHG3), in both TCGA and ICGC databases as the candidate key lncRNAs for subsequent analysis. Furthermore, we explored the relationship between key lncRNAs and the immune infiltration patterns, tumor microenvironment signature, chemosensitivity, tumor neoantigen burden, and clinicopathological characteristics. Finally, the in vitro assays were used to assess the effect of key lncRNAs on HCC cells. The results contribute to clarify the mechanism of how key lncRNAs to affect progression and the prognosis of HCC patients. Notably, we established an accurate prognostic nomogram based on SNHG1 and SNHG3 along with the clinical data to predict OS in patients with HCC.

## 2. Materials and Methods

### 2.1. Acquisition of the Gene Expression Data and the Clinical Information

All the gene expression data and the clinical annotation of the patients with HCC were obtained from The Cancer Genome Atlas (TCGA)-LIHC (https://portal.gdc.cancer.gov/) and International Cancer Genome Consortium (ICGC)-LIHC (https://dcc.icgc.org/) databases [29]. Ultimately, we downloaded data related to 424 samples including 50 normal and 374 HCC samples from TCGA. Whereas data of 444 samples including 242 normal and 202 HCC samples were downloaded from ICGC. The data downloaded from TCGA includes gene expression profile and the clinical data such as survival time, survival status, gender, age, tumor grade, and TMN stage. While the data downloaded from ICGC includes gene expression profile and the clinical data such as survival time and survival status.

### 2.2. Screening of Prognosis-Related Genes

lncRNAs and m6A expression matrixes were obtained from TCGA and ICGC as mentioned above. Pearson correlation analysis was implemented to screen m6A-related lncRNAs (|*r*| > 0.5 and *p* < 0.001). Then, we filtered differentially expressed m6A-related lncRNAs in HCC group in contrast to the normal control group using R package “limma” based on the criteria of |log2Fold Change| > 1 and *p* < 0.05 [30]. Finally, univariate Cox regression analysis was conducted to identify prognosis-related genes.

### 2.3. Analysis of Immune Cell Infiltration

CIBERSORT is a method to characterize cell composition from gene expression profile [31]. It is also the most commonly used tool to estimate and analyze immune cell infiltration. The relative proportion of 22 immune cells of HCC patients was inferred by using CIBERSORT algorithm base on RNA-seq. The sum of all estimated immune cell scores in each sample is equal to 1. The correlation between the abundance of 22 infiltrative immune cells and the expression level of SNHG1 or SNHG3 was analyzed using spearman analysis, and *p* < 0.05 was considered as statistically significant value.

### 2.4. Evaluation of the Sensitivity of Chemotherapeutic Agents

The sensitivity of chemotherapeutic agents was analyzed based on the data obtained from the largest pharmacogenomics database, i.e., Genomics of Drug Sensitivity in Cancer (GDSC) (https://www.cancerrxgene.org/). The R package “pRRophetic” was used to predict the half-maximal inhibitory concentration (IC50) of samples by ridge regression [32]. The prediction accuracy was evaluated through 10-fold cross-validation based on the GDSC training dataset. To remove the batch effect of “combat,” we applied the default values of all parameters. Then, the expression of the duplicate genes was summarized as mean value.

### 2.5. Gene Set Enrichment Analysis

Gene set enrichment analysis (GSEA) was performed to find out different signal pathways between high- and low-expression groups using “clusterProfiler” and “ggraph” package [33]. Groups were divided according to the median expression of SNHG1 or SNHG3. *p* value of < 0.05 was considered statistically significant.

### 2.6. RNA Extraction and qRT-PCR

Total RNA was isolated by Trizol Reagent (Invitrogen). mRNA was reverse transcribed to cDNA by M-MLV reverse transcriptase (Takara, Japan). qRT-PCR was performed using SYBR Green PCR kit (Takara, Japan). GAPDH was used as endogenous controls. The relative expression of genes was calculated using 2^-*ΔΔ*Ct^ method [34]. The sequences of the primers were as follows. GAPDH-F: 5′-TGAGTACGTCGTGGAGTCCAC-3′, GAPDH-R: 5′-GTGCTAAGCAGTTGGTGGTG-3′, SHNG1-F: 5′-ACACTGGGAGCCAATGAAACA-3′, SHNG1-R: 5′-ACACGAAGTGGAGTTATGGGAAG-3′.

### 2.7. Colony Formation Assay

Colony formation assay was performed to measure cell proliferation ability. 300 Huh 7 or HepG2 cells were seeded in 6-well plates and cultured in complete DMEM medium. Fresh culture medium was replaced every 3 days. After 14 days, the colonies were stained with gentian violet. The colony numbers were counted, and each treatment was performed in triplicate [35].

### 2.8. The Wound Healing and Transwell Invasion Assay

Cells were seeded in 6-well plates until the confluence reached 100%. The wound was scratched with a sterile pipette tip. Then, cells were rinsed with DMEM medium without FBS for twice and cultured in complete DMEM medium. The distance that cells had migrated was photographed at the same position and measured at 0 and 48 hours under an inverted optical microscope (Olympus, Japan) [36].

2 × 10^5^ cells were seeded in the upper chamber of Matrigel-coated 24 well transwell chamber. DMEM with 20% FBS was added to the lower chamber of transwell. After 48 hours incubation, the invaded cells at the lower surface of the filter were counted after crystal violet staining [37].

### 2.9. Apoptosis Assay by Flow Cytometry

Cell apoptosis was detected using Annexin V-FITC Apoptosis Detection Kit (LiankeBio, China) according to the manufacturer's protocol [38]. 1 × 10^6^ ~ 3 × 10^6^ cells were harvested and washed with cold PBS. Subsequently, the cells were incubated with 5 *μ*l of Annexin V-FITC and 10 *μ*l of propidium iodide at room temperature for 5 min. Samples were measured using flow cytometry immediately.

### 2.10. Competing Endogenous RNA (ceRNA) Network Construction

Construction of ceRNA network was based on the interaction of key lncRNAs, microRNAs (miRNAs), and m6A genes [39]. First, the mircode (http://www.mircode.org/) database was used to predict the interaction between lncRNA and miRNA. Second, miRDB (http://mirdb.org/) was employed to predict miRNAs which interact with lncRNA related m6A genes. The interaction pairs of lncRNA-miRNA were then integrated with miRNA-mRNA pairs to establish lncRNA-miRNA-mRNA ceRNA network using Cytoscape version 3.8.2.

### 2.11. Statistical Analysis

All statistical analyses were conducted using the R programming language (version 3.6). All statistical tests were bilateral and a *p* value of < 0.05 was considered to be statistically significant. The statistical significance was ^∗^*p* < 0.05, ^∗∗^*p* < 0.01, and ^∗∗∗^*p* < 0.0001.

## 3. Results and Discussion

### 3.1. Results

#### 3.1.1. Screening of m6A-related differentially expressed lncRNAs in HCC.

**Figure 1:**
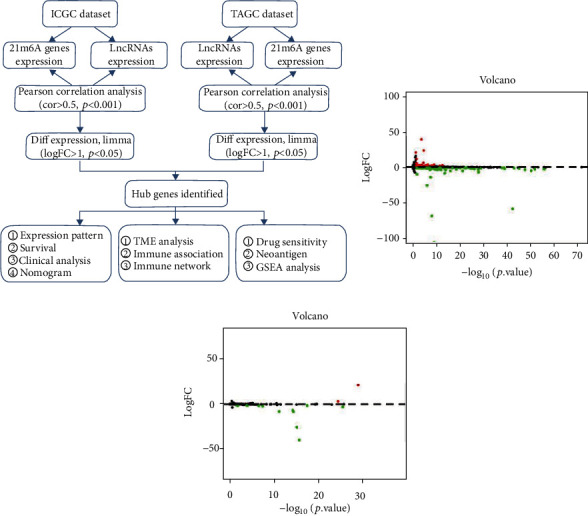
m6A-related differentially expressed lncRNAs in HCC. (a) Study flow chart. (b, c) Volcano plots show the differentially expressed m6A-related lncRNA in TCGA (b) and ICGC (c). Red dot indicates upregulated and green dot indicates downregulated.

In order to identify m6A-related lncRNAs in HCC, we conducted Pearson correlation analysis using lncRNAs and m6A expression matrixes which were downloaded from TCGA and ICGC databases. From TCGA database, we obtained 8777 m6A-related lncRNAs. Whereas, from ICGC database, we obtained 118 m6A-related lncRNAs. Subsequently, we calculated the statistically difference of lncRNA expression level between the normal and HCC groups. In total, 132 (56 upregulated and 76 downregulated) lncRNAs from TCGA (Figure 1(b), supplementary file 1) and 17 (3 upregulated and 14 downregulated) lncRNAs (Figure 1(c), supplementary file 2) from ICGC were identified as differentially expressed m6A-related lncRNAs in HCC.

#### 3.1.2. Prognostic Analysis of the m6A-Related lncRNAs

**Figure 2:**
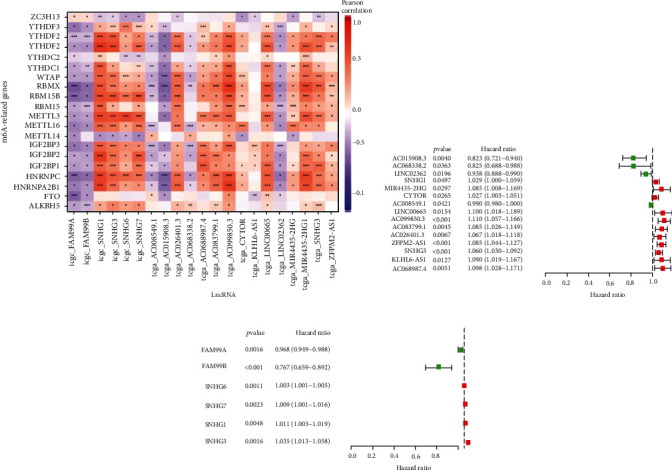
Prognostic values of m6A-related differentially expressed lncRNAs. (a) Heatmap shows m6A-related prognostic lncRNAs from TCGA and ICGC databases. ^∗^*p* < 0.05; ^∗∗^*p* < 0.01; ^∗∗∗^*p* < 0.0001. (b, c) Forest plot shows the hazard ratios of m6A-related differentially expressed lncRNAs from TCGA (b) and ICGC (c). The red box indicates risk factors, and the green box indicates protective factors.

149 differentially expressed m6A-related lncRNAs (132 from TCGA and 17 from ICGC) in HCC were included in univariate Cox regression analysis to evaluate their prognostic roles. Among them, 15 lncRNAs from TCGA and 6 from ICGC were preliminary selected as prognostic genes. The correlation of m6A-related genes and prognostic lncRNAs from both databases is shown in Figure 2(a). As shown in the forest plot (Figure 2(b)), we identified 11 risk factors and 4 protective factors in TCGA geneset. Whereas, from ICGC geneset, we identified 4 risk factors and 2 protective factors (Figure 2(c)). The overlapping geneset among TCGA and ICGC was identified as SNHG1 and SNHG3, which were further recognized as the risk factors along with HR (hazard ratio) > 1 in both genesets (Figures 2(a) and 2(b)).

#### 3.1.3. Association between Key lncRNAs and Tumor Immune Landscape

**Figure 3:**
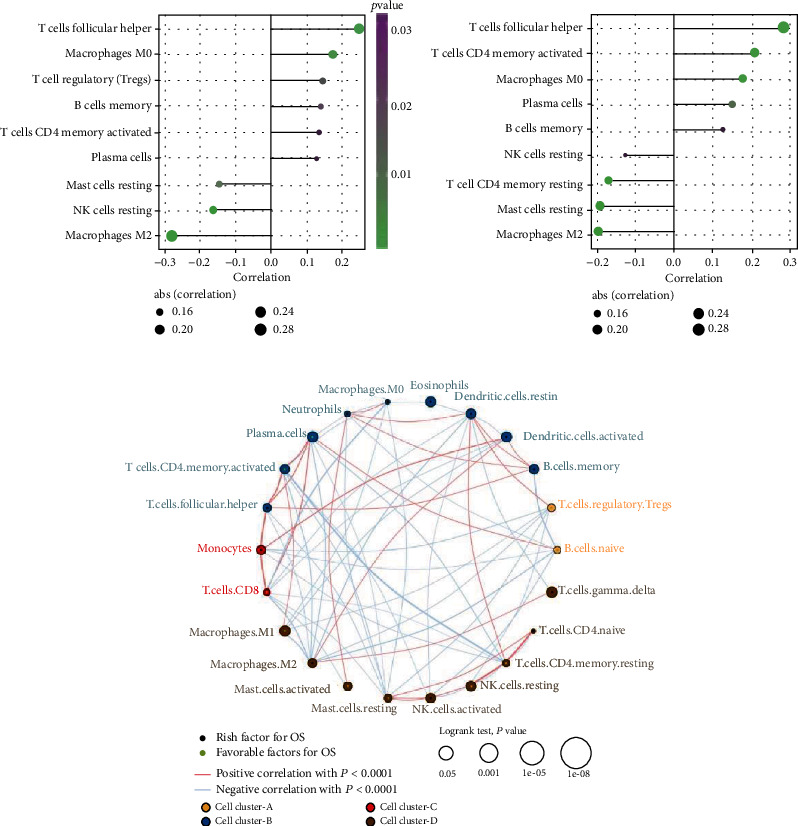
Immune infiltration of landscape and the crosstalk of immune cells. (a, b) Correlation between tumor-infiltrating immune cells and SNHG1 (a) or SNHG3 (b). The size of the dot represents correlation between tumor-infiltrating immune cells and SNHG1/SNHG3. The direction of the horizontal lines indicates positive or negative correlations. The *p* values are indicated by colors. (c) The interaction of different types of immune cells. The size of the circle indicates *p* value calculated by log-rank test. Green dot in the circle indicates favorable factors for OS, and the black dot indicates risk factors for OS. The red lines linking immune cells indicate positive correlation with *p* < 0.01, and the blue lines indicate negative correlation with *p* < 0.01.

Tumor-infiltrating immune cells play a key role in tumor microenvironment. By assessing the infiltration pattern of tumor-infiltrating immune cells, how key lncRNAs affect tumor progression could be further understood. Therefore, we applied CIBERSORT algorithm to RNA-seq data to assess the relative proportions of 22 tumor-infiltrating immune cells. As shown in Figure 3(a), the expression of SNHG1 was positively correlated with T cell follicular helper, macrophages M0, T cell regulatory (Tregs), B cell memory, T cell CD4 memory activated, and plasma cells. Whereas the expression of SNHG1 was negatively correlated with mast cells resting, NK cells resting, and macrophages M2. Further, the expression of SNHG3 was positively correlated with T cell follicular helper, T cell CD4 memory activated, macrophage M0, plasma cells, and B cell memory. Whereas the expression of SNHG3 was negatively correlated with NK cell resting, T cell CD4 memory resting, mast cell resting, and macrophage M2 (Figure 3(b)). Subsequently, according to the correlation with SNHG1 and SNHG3, we divided the immune cells into four clusters: cell cluster A, cell cluster B, cell cluster C, and cell cluster D (Figure 3(c)). Meanwhile, the comprehensive landscape of immune cells interactions and their prognostic significance for liver cancer was analyzed. Results obtained from this analysis identified 12 immune cells (cells memory, T cell CD4 naïve, T cell follicular helper, T cell gamma delta, NK cell activated, monocytes, macrophage M0, macrophages M2, dendritic cell resting, dendritic cell activated, eosinophils, and neutrophils) as risk factors. Whereas we identified 10 (B cells naïve, plasma cells, T cell CD8, T cell CD4 memory resting, T cell CD4 memory activated, T cell regulatory Tregs, NK cell resting, macrophage M1, mast cell resting, and mast cell activated) as favorable factors.

**Figure 4:**
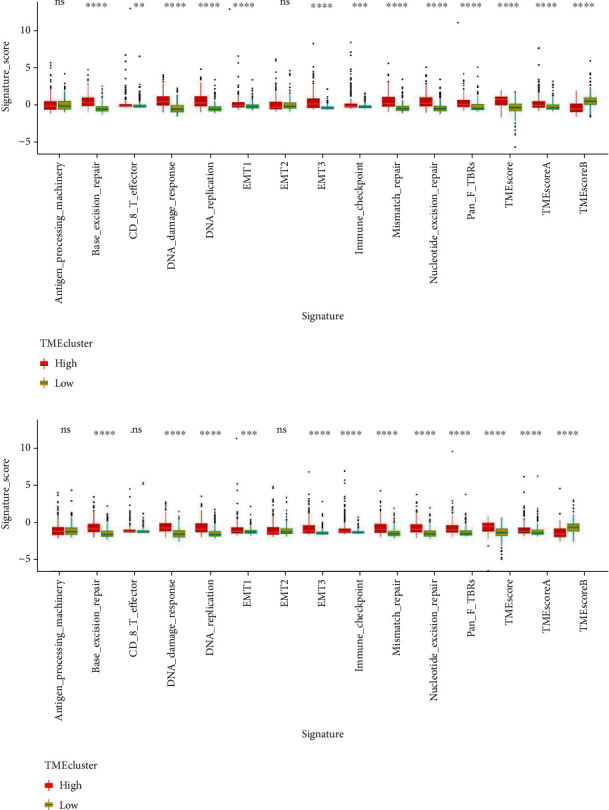
Tumor microenvironment signature. “TMEclusters” was defined according to the expression level of SNHG1/SNHG3. Tumor microenvironment signature was analyzed between high- and low- SNHG1 (a) or SNHG3 (b) expression groups. ns: no significant difference, ^∗^*p* < 0.05; ^∗∗^*p* < 0.01; ^∗∗∗^*p* < 0.0001.

Then, we analyzed the difference of tumor microenvironment signature between high- and low-expression of key lncRNAs groups. “TMEclusters” was identified based on the expression level of SNHG1 or SNHG3. As shown in Figure 4, we identified 12 tumor microenvironment signatures with significant difference between high- and low-expression groups, including base excision repair, CD8 T effector, DNA damage response, DNA replication, EMT1, EMT3, immune checkpoint, mismatch repair, nucleotide excision repair, pan F TBRs, TMEscore, TMEscoreA, and TMEscoreB.

These results suggested that SNHG1 and SNHG3 expression affected immune infiltration and the crosstalk among immune cells probably played an important role in the formation of immune infiltration characterization of tumor microenvironment.

#### 3.1.4. Chemosensitivity Associated with Key lncRNAs

**Figure 5:**
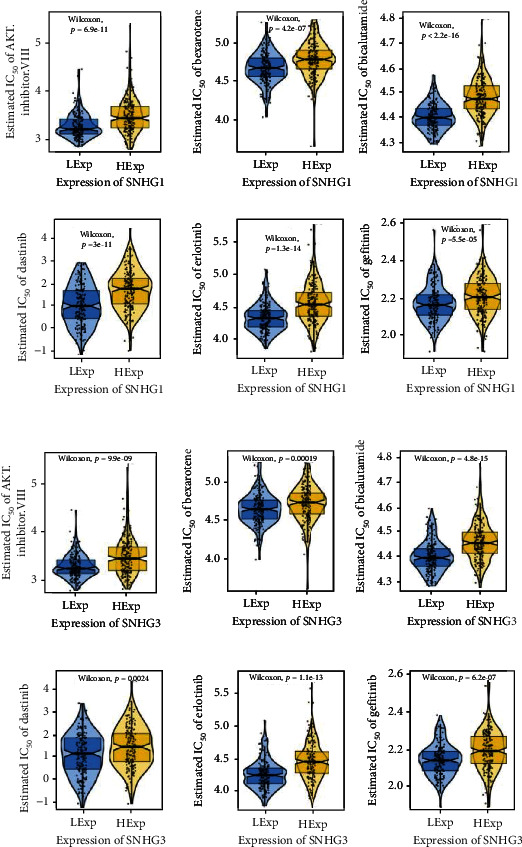
Chemosensitivity analysis of in HCC patients with high- or low- expression of SNHG1 or SNHG3. The estimated IC50 for AKT inhibitor VII, bexarotene, bicalutamide, dasatinib, erlotinib, and gefitinib in high- and low- SNHG1 (a) or SNHG3 (b) expression groups. LExp: low-expression; HExp: high-expression. The statistical difference between two groups was compared through the Wilcoxon test. *p* values were shown in the figure.

Considering the fact that chemotherapy is commonly used in the comprehensive treatment for liver cancer, we assessed the relationship between key lncRNAs expression and the sensitivity of six chemotherapeutic agents: AKT inhibitor VII, bexarotene, bicalutamide, dasatinib, erlotinib, and gefitinib (Figures 5(a) and 5(b)). According to the median expression level of SNHG1 or SNHG3, all the tumor samples were divided into two groups: high-expression and low-expression groups. Samples with expression level higher than the median were divided into high-expression groups and lower than the median were divided into low-expression groups. We used R package “pRRophetic” to estimate the IC50 of each sample. As observed in the result, significant difference in the IC50 existed between high- and low-expression groups for all the 6 chemotherapeutic agents. Samples with low expression of SNHG1 or SNHG3 were probably more sensitive to AKT inhibitor VII, bexarotene, bicalutamide, dasatinib, erlotinib, and gefitinib.

#### 3.1.5. Tumor Neoantigen Burden in High- and Low-SNHG1/SNHG3 Groups

**Figure 6:**
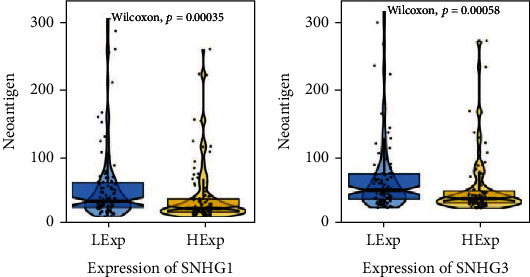
Correlation of tumor neoantigen and the expression of SNHG1 and SNHG3. The difference of tumor neoantigen between high- and low-SNHG1 (a) or SNHG3 (b) expression was analyzed through the Wilcoxon test. LExp: low-expression; HExp: high-expression. *p* values were shown in the figure.

Immunotherapy has been considered as a major breakthrough in cancer therapy, and tumor neoantigen burden is closely associated with the efficacy of immunotherapy. As shown in our results, the expression of key lncRNAs was correlated with tumor neoantigen burden. Tumor neoantigen burden was higher in the low-expression of SNHG1 or SNHG3 group than that in high group (Figures 6(a) and 6(b)).

#### 3.1.6. Function Annotation of Key lncRNAs

**Figure 7:**
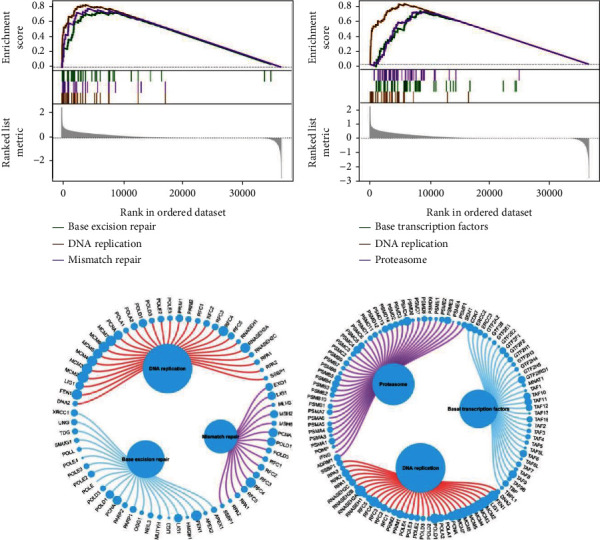
GSEA of pathway enrichment in high SNHG1 or SNHG3 group. GSEA revealed pathways enriched in high SNHG1 (a) or SNHG3 (b) group. Molecules involved in the enriched pathways in high SNHG1 (c) or SNHG3 (d) group.

To investigate the potential mechanism of prognostic genes affecting the tumor progression, we carried out GSEA using data from TCGA and ICGC. As indicated in Figure 7(a), the high-expression of SNHG1group was significantly associated with pathways including base excision repair, DNA replication, and mismatch repair. Meanwhile, pathways including basal transcription factors, DNA replication, and proteasome were significantly enriched in high-expression of SNHG3 group (Figure 7(b)). Molecules involved in the enriched pathways were shown in Figures 7(c) and 7(d), respectively. These results indicated that the alteration of SNHG1 or SNHG3 expression may affect the tumor progression through the pathways mentioned above.

#### 3.1.7. Association between Key lncRNAs and the Clinicopathological Characteristics

**Figure 8:**
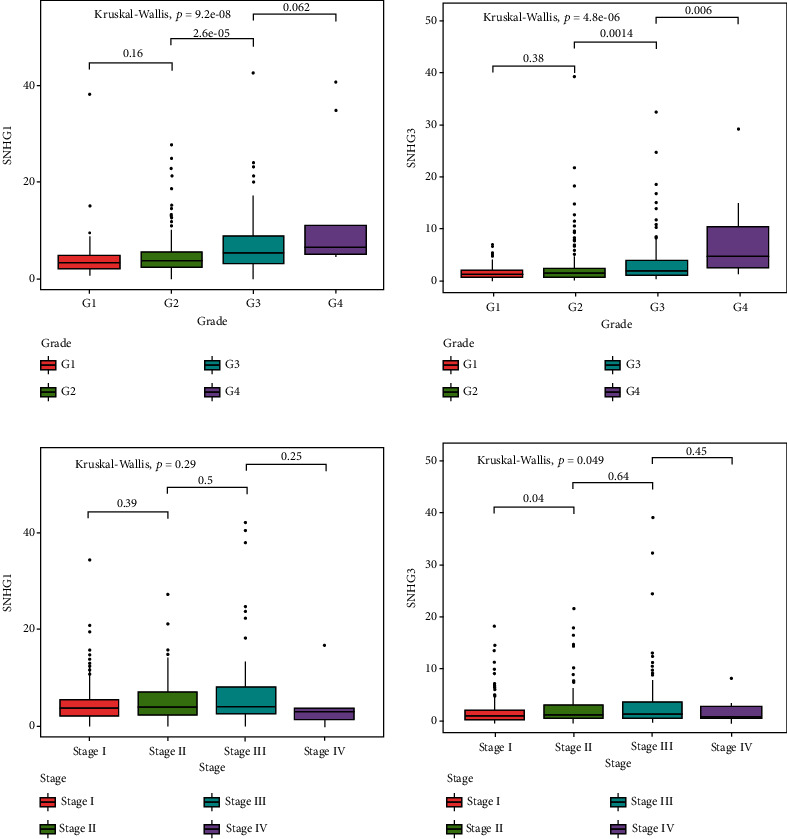
Expression pattern of SNHG1 and SNHG3. SNHG1 (a) and SNHG3 (b) expression of different grades. SNHG1 (c) and SNHG3 (d) expression of different stages.

Subsequently, we analyzed the expression level of key lncRNAs in different histological grade and pathological stage tumor samples. As observed in the results, the expression of SNHG1/SNHG3 was higher in advanced high tumor grade (Figures 8(a) and 8(b)). Additionally, no significant difference was observed between the clinical stages for the expression level of SNHG1 (Figure 8(c)). However, we observed significantly upregulated expression of SNHG3 in stage II compare with stage I (Figure 8(d)). These results suggest that aberrant upregulation of SNHG1 and SNHG3 is closely related to clinicopathological characteristics of HCC.

#### 3.1.8. Establishment of a Prognostic Nomogram for HCC Patients

**Figure 9:**
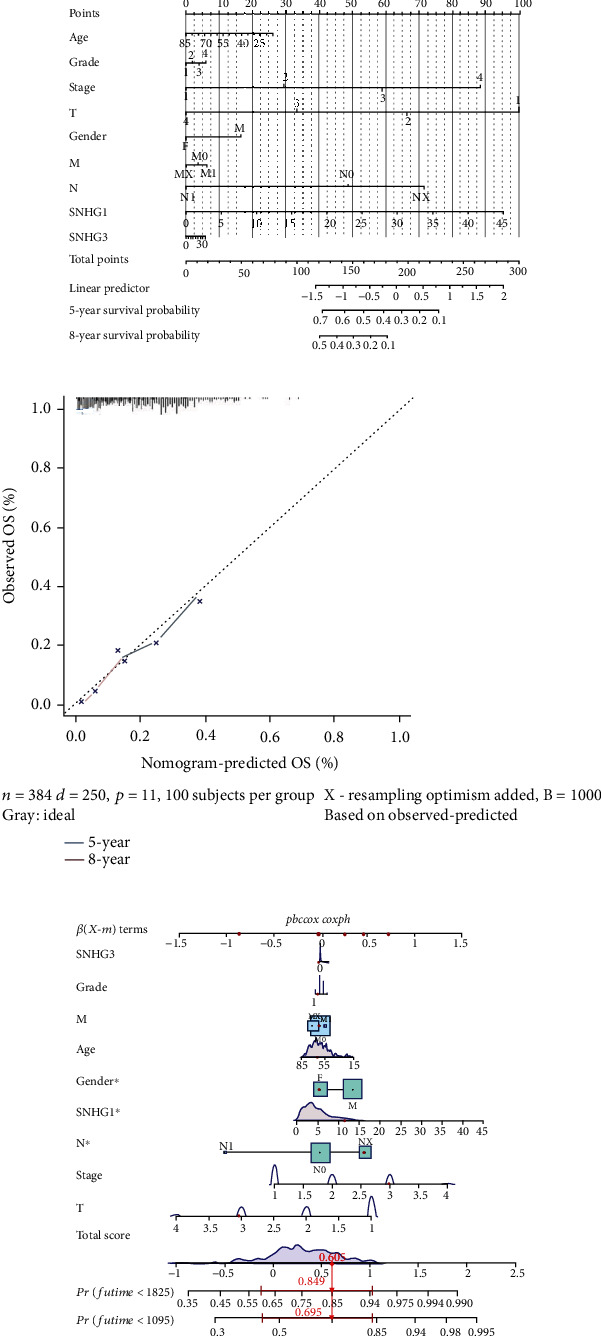
Nomogram for the OS prediction of HCC patients. (a) The nomogram based on SNHG1 and SNHG3 combined with age, grade, stage, T, gender, M, and N to predict 5-year and 8-year OS of HCC patients. (b) The calibration curve for the predicted 5-year and 8-year OS of HCC patients according to the nomogram. The *x*-axis indicates the predicted OS, and the *y*-axis indicates the observed OS. (c) Construction of the Cox proportional-hazards model and SNHG1, SNHG3, grade, M, age, gender, N, stage, and T were included in the model.

In order to create a clinically applicable quantitative tool to predict the OS of liver cancer patients, a prognostic nomogram was established based on the age, histological grade, pathological stage, gender, and key lncRNAs (Figure 9(a)). The results suggested that the expression of SNHG1 had a prominent contribution to the overall survival score while SNHG3 contributed less. Similarly, the analysis of Cox proportional-hazard model (coxph) showed the results in accordance with that of the nomogram (Figure 9(c)). Additionally, the nomogram systematically predicted the 5- and 8-year overall survival of liver cancer patients. Furthermore, the calibration curve revealed a reasonable concordance between observed and predicted OS (Figure 9(b)). These results suggested that the nomogram could predict the 5- or 8-year OS of liver cancer patients with good accuracy.

#### 3.1.9. Construction of the ceRNA Network

**Figure 10:**
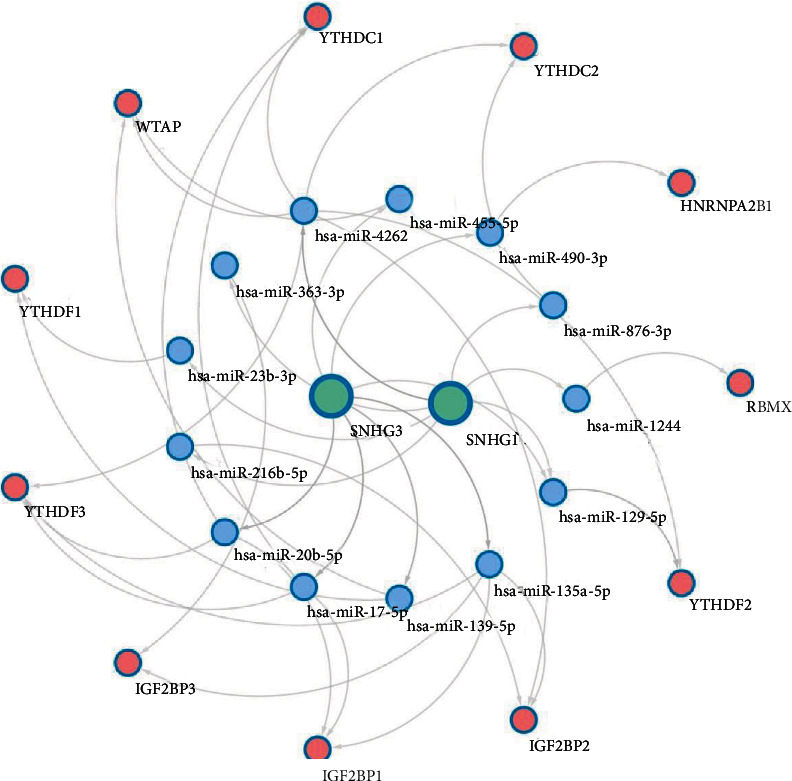
Key lncRNAs based ceRNA network. Bluish green circles indicate lncRNAs, blue circles indicate miRNAs, and pink circles indicate m6A genes.

lncRNAs can act as miRNAs sponges to sequester miRNAs away and further regulate mRNAs. To better elucidate the regulatory mechanisms of key lncRNAs in HCC, we constructed the ceRNA network. Mircode database was used to predict the targets of the key lncRNAs, and 149 lncRNA-miRNA pairs were obtained. miRDB database was used to predict miRNAs which interact with the key lncRNAs related m6A genes, and 2731 miRNA-mRNA pairs were obtained. After separately identifying the lncRNA-miRNA and miRNA-mRNA pairs, the overlapped interaction pairs were used to construct the ceRNA network. Finally, 11 lncRNA-related m6A genes and 13 miRNAs were included in the ceRNA network (Figure 10).

#### 3.1.10. Effects of Key lncRNAs on Behaviors of HCC Cells

**Figure 11:**
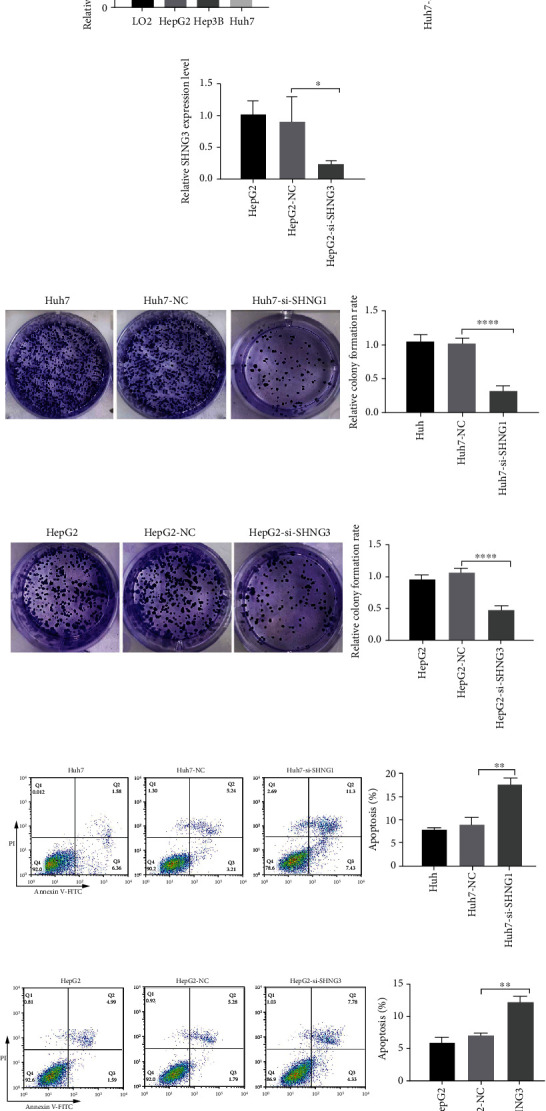
SHNG1 and SHNG3 inhibit apoptosis and promote proliferation of HCC cells. (a) Expression of SHNG1 in LO2, HepG2, Hep3B, and Huh7 cell lines was measured by qRT-PCR. (b) siRNA knockdown efficiency was confirmed by qRT-PCR in Hun7 cells. Huh7: Huh7 cells without transfection; Huh7-NC: Huh7 cells transfected with negative control (NC); Huh7-si-SHNG1: Huh7 cells transfected with si-SHNG1. (c) siRNA knockdown efficiency was confirmed by qRT-PCR in HepG2 cells. HepG2: HepG2 cells without transfection; HepG2-NC: HepG2 cells transfected with negative control; HepG2-si-SHNG3: Huh7 cells transfected with si-SHNG3. (d, e) Colony formation assay was used to assess the effect of siSHNG1 in Huh7 cells and the effect of siSHNG3 in HepG2 cells. (f, g) Flow cytometry analysis of Annexin V-FITC/PI staining was used to assess the number of apoptotic cells in each treatment in Huh7 and HepG2 cells. ^∗^*p* < 0.05; ^∗∗^*p* < 0.01; ^∗∗∗^*p* < 0.0001.

To further confirm the expression pattern of key lncRNAs in HCC cell lines, we measured the relative expression level of SNHG1 in LO2, HepG2, Hep3B, and Huh7 cell lines by qRT-PCR. As shown in Figure 11(a), SNHG1 was significantly upregulated in HCC cell lines compared with that of human immortalized hepatocyte cell line LO2. In addition, the relative expression level of SNHG3 in HCC cell lines was determined in our previous study. The expression level of SNHG3 was higher in HCC cell lines than that in LO2 [40]. In conclusion, both SNHG1 and SNHG3 were upregulated in HCC cell lines. The expression level of SNHG1 was highest in Huh7, and the expression level of SNHG3 was highest in HepG2.

To study the role of key lncRNAs in HCC cells, SNHG1 was knocked down in Huh7, and SNHG3 was knocked down in HepG2 by small interfering RNA (siRNA), separately (Figures 11(b) and 11(c)). The colony formation assays were used to assess the role of key lncRNAs in HCC proliferation. The results showed that suppression of SNHG1 by siRNA inhibited the proliferation of Huh7 and suppression of SNHG3 inhibited the proliferation of HepG2 (Figures 11(d) and 11(e)). Cell apoptosis was detected using flow cytometry analysis of Annexin V-FITC/propidium iodide (PI). The results showed that knocking down of SNHG1 or SNHG3 promoted cell apoptosis in Huh7 or HepG2 (Figures 11(f) and 11(g)). These results suggested that SNHG1 and SNHG3 could inhibit apoptosis and promote proliferation of HCC cells.

**Figure 12:**
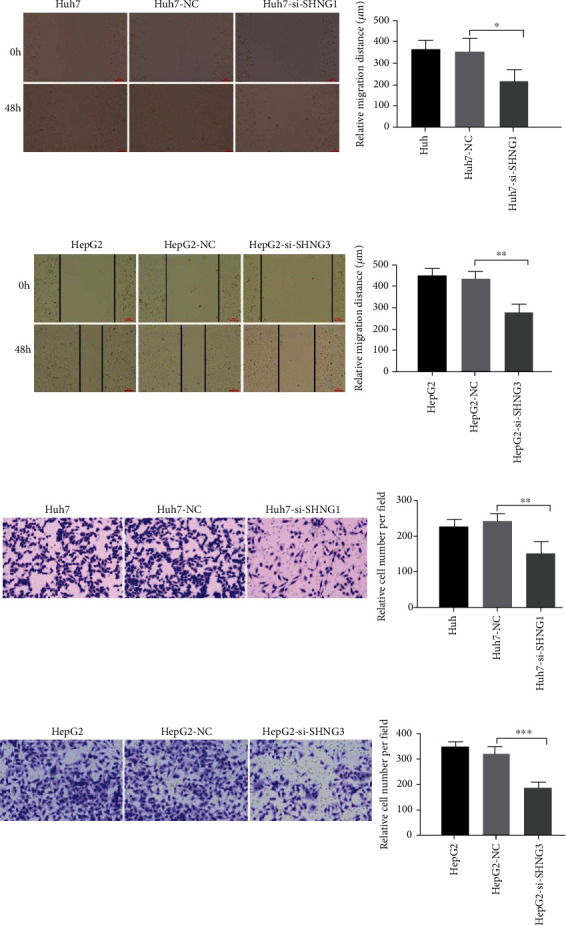
SHNG1 and SHNG3 promote migration and invasion ability of HCC cells. (a, b) The effect of siSHNG1 or siSHNG3 on cell migration ability was determined by wound healing assay in Huh7 or HepG2 cells. Scar bars: 100 *μ*m. (c, d) The effect of siSHNG1 or siSHNG3 on cell invasion ability was assessed by transwell assay in Huh7 or HepG2 cells.

To further investigate the effects of key lncRNAs on migration and invasion of HCC cells, the wound healing assay and transwell assays were performed. The result of the wound healing assay showed that cells treated with siSNHG1 or siSNHG3 exhibited slower rate of migration of HCC cells than the NC transfected or the untransfected cells (Figures 12(a) and 12(b)). Moreover, the result of transwell assays indicated that the reduction of SNHG1 or SNHG3 inhibited the invasion ability of HCC cells (Figures 12(c) and 12(d)).

In conclusion, the results indicate that SNHG1 and SNHG3 promote the proliferative, migratory, and invasive abilities of HCC cells.

### 3.2. Discussion

The overall prognosis for HCC patients is poor, with a 5-year survival rate of less than 5% in underdeveloped areas [41]. Therefore, prognostic signature which can distinguish high-risk and low-risk patients is essential for determining optimal therapeutic strategies and improving survival outcomes of HCC patients. Emerging evidence has demonstrated that m6A-related genes could be prognostic markers for predicting the survival time of patients [42–44]. In recent years, the role of m6A related lncRNAs has been recognized profoundly in HCC [45, 46]. And studies in terms of prognosis of HCC patients were also accumulated. As an instance, high expression level of LINC00958 which is mediated by m6A modification predicts poor overall survival of HCC patients independently [47]. Moreover, studies using a comprehensive informatics analysis based on data from TCGA database revealed that m6A methylated lncRNA was closely related to the prognosis of HCC [48–50]. However, more effective and specific prognostic biomarkers still needed.

To screen new m6A-related lncRNA signature which is responsible for prognosis of HCC, a total of 576 HCC patients and 292 normal cases were included in our study for a comprehensive bioinformatics analysis. From the results, two m6A-related lncRNAs, namely, SNHG1 and SNHG3 were found to be differentially expressed in HCC and act as negative prognostic factors in both TCGA and ICGC databases. Small nucleolar RNA host genes (SNHGs) have been recognized as important regulators in the development and prognosis of multiple cancers. Previous studies have shown that SNHG1, SNHG3, SNHG5, SNHG6, SNHG8, SNHG16, and SNHG20 were all upregulated in HCC and associated with process of carcinogenesis such as cell proliferation, invasion, and metastasis [51–60]. Among them, SNHG1, SNHG3, SNHG16, and SNHG20 were demonstrated to be associated with poor prognosis [51, 56, 59, 60]. Additionally, SNHG16 was linked to chemoresistant, while SNHG3 facilitated sorafenib resistance [54, 55]. In the present study, we identified SNHG1 and SNHG3 as significantly upregulated m6A-related lncRNAs in HCC samples compared to the normal cases. This suggested their probable role in HCC carcinogenesis. The in vitro experiments revealed that SNHG1 and SNHG3 were upregulated in HCC cell lines compared to LO2, and they promoted the proliferative, migratory, and invasive abilities of HCC cells. These results indicate that SNHG1 or SNHG3 promotes the development and progression of HCC, which is consistent with previous studies in HCC or other cancer types [61–63]. ceRNA network was established to explore the regulatory mechanisms of SNHG1 and SNHG3. 13 miRNAs were included in the ceRNA network and associated with both SNHG1 and SNHG3. Among them, miR-139-5p was reported to interact with SNHG3 in gastric cancer [62], clear cell renal cell carcinoma [64], ovarian cancer [65], and HCC [66]. SNHG1 sponged miR-216b-5p to promote cell growth and migration in serous epithelial ovarian cancer [67] and promote tumor angiogenesis and growth in breast cancer [68]. These findings provide further confirmation that SNHG1 and SNHG3 affect HCC cell behaviors through miRNAs.

Furthermore, univariate Cox regression analysis revealed SNHG1 and SNHG3 acted as negative prognostic factors. The results obtained from the present study were consistent with the previous reports which showed linkage of SNHG1 and SNHG3 to poor prognosis. Moreover, the results of further analysis suggest the low expression of SNHG1/SNHG3 corresponding to more sensitive to AKT inhibitor VII, bexarotene, bicalutamide, dasatinib, erlotinib, and gefitinib. These results indicate that high expression of SNHG1/SNHG3 can lead to drug resistant. The results are in better agreement with reports related to SNHG and chemoresistant. Previous studies reported that increased SNHG3 expression was correlated with poor prognosis and sorafenib resistance partly through inhibiting miR-128 signalling [55]. The study carried out by Ge et al. showed contribution of SNHG1 in cisplatin resistance related to nonsmall cell lung cancer [69]. In another report, high expression of SNHG3 was positively associated with cisplatin resistance in gastric cancer cells [70].

The immune response in the microenvironment has been identified to participate actively in the progression and recurrence of cancer, which contributed to the adverse outcomes of patients. Tumor-infiltrating cells, which are the vital part of tumor microenvironment, positively correlated with malignancy progression and unfavorable prognosis of tumor. Previous studies have revealed that m6A modification played an undeniably important role in the formation of tumor microenvironment characterizations [71]. In the present study, T cell follicular helper, macrophage M0, B cell memory, and plasma cells were positively correlated with both m6A-related lncRNAs SNHG1 and SNHG3. Whereas mast cell resting, NK cell resting, and macrophage M2 were negatively correlated with SNHG1 and SNHG3. SNHG1 and SNHG3 may affect the immune cell infiltration patterns and the crosstalk among immune cells, thus shedding light on how SNHG1 and SNHG3 interact with tumor microenvironment.

Both SNHG1 and SNHG3 were observed with correlation to treatment decisions and prognosis of patients with cancers [55, 57, 60]. Further, we analyzed the expression level of SNHG1 and SNHG3 in tumors with different grades and stages. The expression level of both SNHG1 and SNHG3 was related to advanced tumor grade. Further, we observed significantly higher expression level of SNHG3 in stage II than in stage I. SNHG1 was reported to be associated with advanced pathological stage in multiple cancers [61, 72–74]. However, the expression of SNHG1 was not associated with clinical stage of HCC patients in our study. But the expression level of SNHG3 was closely associated with advanced tumor grade and stage, which is consistent with those of previous reports in HCC or other cancers [62, 63, 75, 76]. These results revealed that the expression level of SNHG1 and SNHG3 could serve as candidate biomarkers for optimizing treatment strategy and predicting prognosis of HCC patients.

## 4. Conclusions

In conclusion, aberrant upregulation of m6A-related lncRNA SNHG1 and SNHG3 was found closely related with worse prognosis of HCC patients and was of great significance in predicting the overall survival of HCC patients.

## Data Availability

The datasets generated and analyzed are available in the TCGA, ICGC, and GDSC database.

## Supplementary Materials

Supplementary 1 Supplementary file 1 differentially expressed m6A-related lncRNAs in CGA.

Supplementary 2 Supplementary file 2: differentially expressed m6A-related lncRNAs in ICGC.
